# Robustness of Auditory Teager Energy Cepstrum Coefficients for Classification of Pathological and Normal Voices in Noisy Environments

**DOI:** 10.1155/2013/435729

**Published:** 2013-05-28

**Authors:** Lotfi Salhi, Adnane Cherif

**Affiliations:** Signal Processing Laboratory, Physics Department, Sciences Faculty of Tunis, University of Tunis ElManar, 1060 Tunis, Tunisia

## Abstract

This paper focuses on a robust feature extraction algorithm for automatic classification of pathological and normal voices in noisy environments. The proposed algorithm is based on human auditory processing and the nonlinear Teager-Kaiser energy operator. The robust features which labeled Teager Energy Cepstrum Coefficients (TECCs) are computed in three steps. Firstly, each speech signal frame is passed through a Gammatone or Mel scale triangular filter bank. Then, the absolute value of the Teager energy operator of the short-time spectrum is calculated. Finally, the discrete cosine transform of the log-filtered Teager Energy spectrum is applied. This feature is proposed to identify the pathological voices using a developed neural system of multilayer perceptron (MLP). We evaluate the developed method using mixed voice database composed of recorded voice samples from normophonic or dysphonic speakers. In order to show the robustness of the proposed feature in detection of pathological voices at different White Gaussian noise levels, we compare its performance with results for clean environments. The experimental results show that TECCs computed from Gammatone filter bank are more robust in noisy environments than other extracted features, while their performance is practically similar to clean environments.

## 1. Introduction

In the objective support of the analysis and the selection of vocal and voice diseases, the automatic evaluation of voice quality based on acoustic analysis stays an efficient tool. In the speech pathology field, on which this work focuses, pathological voices can be evaluated using two approaches that are perceptual analysis and objective analysis. The analysis of pathological voice is a hot topic that has received large attention. There are several medical diseases that harmfully affect our human voice like laryngitis, laryngeal atypia and early/advanced cancer, Reinke's edema, RRP-papillomatosis, spasmodic dysphonia, vocal fold granuloma, vocal fold paresis/paralysis, voice dysfunction in neurological disorders (stroke, Parkinson's disease, benign essential tremor, amyotrophic lateral sclerosis, myasthenia gravis, multiple sclerosis), and pediatric voice disorders. The analysis of the voice disorder stays essentially clinic [1, 2]. The clinician can use the available apparatus for identification of pathological voice. It is usually made by laryngoscopical exams, which are considered invasive, and requires an expert analysis of numerous human speech signal parameters. Automatic analysis of pathological voices has its advantages, such as having its quantitative and noninvasive nature. Furthermore, it allows the detection and supervising of vocal system diseases and reducing analysis charge and time. Based on the voice of a patient, the goal of pathological voice classification is to make a decision whether it is normal or pathological. Successful pathological voice classification will enable an automatic noninvasive device to diagnose and analyze the voice of the patient.

In the current literature, the majority of approved researches in this area have been oriented to the study of acoustic parameters perturbation measurements and noise. The features used are often extracted from the audio data for voice pathology analysis including the fundamental frequency (*F*_0_), jitter, shimmer, Mel-Frequency Cepstral Coefficients (MFCC), signal-to-noise ratios (SNR), Harmonic-to-Noise Ratios (HNR), and High Order Statistics parameters (HOS) [3]. However, the research for a more detailed and representative acoustic analysis of pathological voice signals is still a promising area. Also, the techniques based on the description of the spectral components to detect the disorder glottal activity have been shown to be consistent in the detection of pathological voices [4].

Regardless of recent advances in the state of the art of automatic classification of pathological and normal voices, identification of pathological voices in noisy conditions remains an open research problem [5]. In general robust identification of pathological voices is an important research area. Performance of all speech parameters deployed in the field often degrades due to adverse and unexpected environmental conditions. Most approaches that have been used in the literature for improving the voice disorders identification in noisy environments mainly fall into three categories: acoustic model adaptation algorithms, speech enhancement algorithms, and robust feature extraction algorithms [6]. In this study, we focus on the topic of robust feature extraction. So, we propose to use the auditory Teager energy feature set for parameterization of voice signal. This proposition is motivated by speech perception consideration that is based on the human perception models and the nonlinear Teager-Kaiser operator that provide a good estimation of the “real” energy of the source of a resonance signal [7, 8]. It is for this reason that this parameter was used recently for identification of pathological voices. 

In this study, a parametric analysis based on auditory Teager energy is employed to discriminate pathological voices of speakers affected by different vocal pathologies in noisy environments. In addition to the TECCs performances, the robustness of the proposed system is motivated by the use of human perception models which is a filter bank of one of the three auditory systems: Gammatone or Mel scale triangular. The filter bandwidths are proportional to the auditory Equivalent Rectangular Bandwidth (ERB) function as described in [9–11]. However, the Multilayer Neural Network has been generally used because there is no need to think about the details of the mathematical models of the data and it is reasonably easy to train and has produced a good pathological recognition performance [12, 13]. We admit that a comparison with other feature classification results is needed to evaluate the performance of the proposed feature. In this paper, the MLP method was used to classify the mixed voiced dataset. The proposed features labeled auditory Teager Energy Cepstrum Coefficients (TECCs) are evaluated on tasks of classification of pathological and normal voices in noisy and clean condition. Then, a comparison of the performance of different used features was performed in order to show that it is the most robust in noisy environment. Note that the robustness is shown in terms of correct classification rate (CCR) accuracy.

## 2. Materials and Methods

### 2.1. Identification of Pathological Voices System

**Figure 1:**
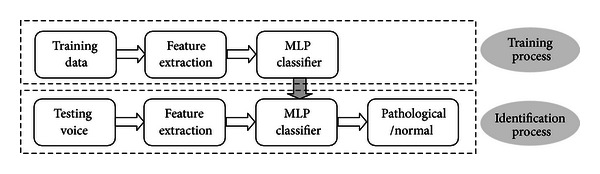
Block diagram of the proposed system.

The proposed approach for the task of automatic classification of pathological and normal voices in noisy environments essentially consists of two parts: feature extraction and classification.  Figure 1 illustrates the block diagram of the proposed system.

In the training process, we train the MLP classifier with feature model using the training voice data. We use the supervised training algorithm. So, we give each speech sample with the corresponding nature class label. Then the MLP classifier will be saved with all his specific parameters. In the identification part, the input of this system is  .wav files, which come from the testing database or from the real-time speech.

### 2.2. Corpus

One corpus comprises four sentences produced by 62 normophonic speakers (35 male and 27 female) and 50 dysphonic speakers (28 male and 22 female). The mean age of the selected patients was 53 years (range, 32 to 75 years). Also, the mean age of the volunteers' normophonic speakers working in or around the laboratory was 47 years (range, 28 to 82 years). The patients had been diagnosed on the base of a clinical examination at the ENT Department of the Rabta Hospital in Tunis, Tunisia [14, 15]. Continuous speech samples from patients with a wide variety of organic, neuralgic, traumatic, and psychogenic voice disorders, as well as 62 normal subjects are included. The pathologies had been determined as follows: vocal cord palsy, vocal edema, vocal polyps, vocal nodules, vocal cysts, chronic laryngitis, glottic cancer, Parkinson's, and Alzheimer's. Subjects were instructed to produce four standardized Arabic sentences at a comfortable pitch and volume as naturally as possible without overacting. 

A second corpus comprises sustained vowels “*a*,” including onsets and offsets, and four French sentences produced by 22 normophonic or dysphonic speakers (10 male and 12 female speakers) [16, 17]. The corpus includes 20 adults (from 20 to 79 years), one boy aged 14 years and one girl aged 10 years. Five speakers are normophonic, the others are dysphonic. The dysphonic speakers were patients of the laryngology department of a university hospital in Brussels, Belgium. The disordered voices range from mildly deviant to very deviant. The pathologies were diagnosed as follows: dysfunctional dysphonia, bilateral nodule, polyp on the left vocal fold, edema of the vocal folds, mutational disorder, dysphonia plica ventricularis, and unilateral vocal fold paralysis. The sentences are referred to as *S*_1_, *S*_2_, *S*_3_, and *S*_4_, respectively. They have the same grammatical structure, the same number of syllables, and roughly the same number of resonants and plosives. Sentences *S*_1_ and *S*_2_ are voiced by default, whereas *S*_3_ and *S*_4_ include voiced and unvoiced segments. Speech signals have been recorded at a sampling frequency of 48 kHz. The recordings were made in an isolated booth by means of a digital audio tape recorder (Sony TCD D8) and a head-mounted microphone (AKG C41WL). The recordings have been transferred from the DAT recorder to computer hard disk via a digital-to-digital interface. Silent intervals before and after each recording have been removed by manual segmentation.

### 2.3. MLP Classifier

In the last years, neural networks are among the popular signal-processing technologies. In speech processing, neural networks supply as pattern classifiers and as nonlinear adaptive filters. The most popular example of neural network in many tasks of pattern recognition is the multilayer perceptron (MLP) [13]. In the MLP which has one layer or more, the neurons of each layer are interconnected with each other by weights. The activation function of each layer of neurons is a specific mathematical function that allows the neuron to generate an output for the next layer. This activation function is calculated based on the sum of the product between the input vector and the synaptic weights of each unit. Generally, the MLP is trained using the descent gradient method [13].

The trained neural networks are an essential step that can allow the system to learn the prospective interaction between voice quality indices and their corresponding classes. Also it can provide an output representing the definite category for each of voice class indices, whereas the testing step is used to verify the classification ability of the proposed neural networks and thus deduce the CCR of the used speech feature.

**Figure 2:**
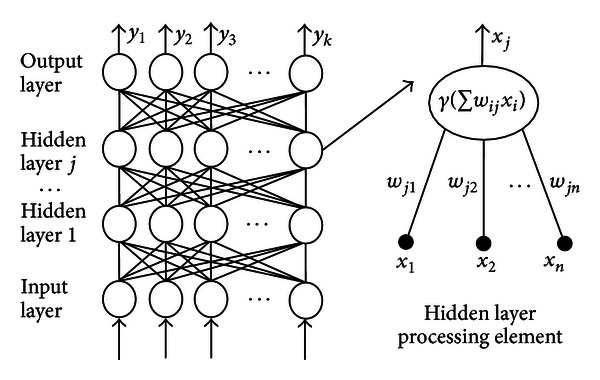
General schematic of a neural network (MLP).

Figure 2 gives you an idea about the general schematic of a neural network (MLP) and to an artificial neuron.

### 2.4. Feature Extraction

**Figure 3:**
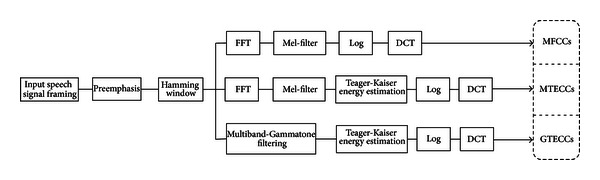
Block diagrams of the extraction of MFCC, MTECC, and GTECC features.

The speech signal has many acoustic features which reflect the pathological voices characteristics. In the research domain of classification of pathological and normal voices, the importance of feature extraction is how to extract and select the most pertinent speech features with which most voice pathologies could be identified. Different parameters where chosen at the input of the neural networks such as speech rate, energy, pitch, formant, Linear Prediction Coefficients (LPC), Linear Prediction Cepstrum Coefficients (LPCC), Mel-Frequency Cepstrum Coefficients (MFCCs) and their derivative. The type of each parameter depends on its method of extraction. In this study, our objective is to introduce new speech features that are more robust in classification of pathological and normal voices in noisy environments. We propose a robust speech feature which is based on the combination of the Teager-Kaiser energy cepstrum and an auditory (Gammatone) filter bank (Figure 3).

We investigate the robustness and compare the performance of the proposed GTECC features to that of MFCCs and MTECCs by artificially introducing different levels of white noise to the speech signal and then computing their correct classification rate. 

As illustrated in Figure 3, it can be seen that one of the main dissimilarity between MTECC and GTECC is the set of filters used in the extraction. In fact, triangular filter bank equally spaced in the Mel scale frequency axis is used to extract MTECCs features, while in GTECC, the Gammatone filter bank are used. For instance, on the computing of MTECC and GTECC features, the speech energy is estimated through Teager-Kaiser energy operator (TEO).

### 2.5. Theoretical Framework

#### 2.5.1. Mel-Frequency Cepstrum Coefficients (MFCCs)

The MFCC features are a parameters family that may be deducted either using a parametric approach resulting from linear predictive coefficients or using a nonparametric approach based on the Fast Fourier Transform (FFT). In our study we use the nonparametric approach because it allows modelling of the effects induced by the presence of pathology over the excitation (vocal folds) and the system (vocal tract). In recent literature, the MFCC features are mostly used for speech recognition and it presents an excellent performance in this task. Their success occurs from the use of perceptually based Mel-spaced filter bank processing of the Fourier transform and the particular robustness and flexibility that can be achieved using cepstral analysis [18].

Consequently, we derive the filter bank values by cater-cornered; we multiply the *K* triangular filter bank weighting function by the NFFT magnitude coefficients and then we collect each filter triangle results.

**Figure 4:**
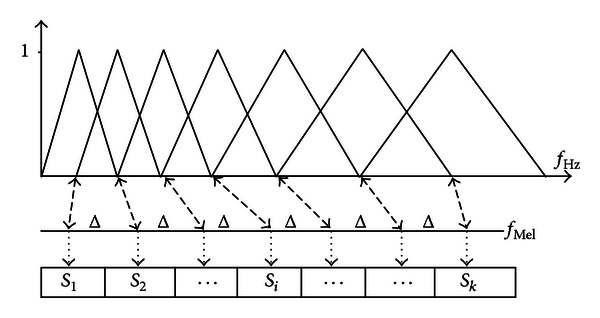
Principle of Mel scale filter bank.

In order to reflect the human hearing logarithmic compression, we usually take the log of the filter bank output. As indicated by Figure 4, the spacing of the triangle filters bank centres occurs according to the Mel scale defined by the following [19, 20]:

(1) $$\begin{array}{c} f_{\text{Mel}}=2595\;\log_{10}\left(1+\frac{f_{\text{Hz}}}{700}\right). \end{array}$$

Finally and based on the Discrete Cosine Transform (DCT), we apply the cepstral analysis which consists in converting the log filter bank spectral values into cepstral coefficients as shown in

(2) $$\begin{array}{c} C_{n}=\;\sum_{i=1}^{K}\log_{10}\left(S_{i}\right)\cos\left[n\left(i-\frac{1}{2}\right)\frac{\pi }{K}\right], \end{array}$$

where *i* = (1, 2, …, *K*), *K* represents the number of the Mel bands in the Mel scale, *n* = (1, 2, …, *N*), *N* being the number of MFCCs extracteds and *S*_*i*_ is the short-time Fourier transform (STFT) of the input discrete signal.

#### 2.5.2. Teager-Kaiser Energy Operator (TEO)

**Figure 5:**
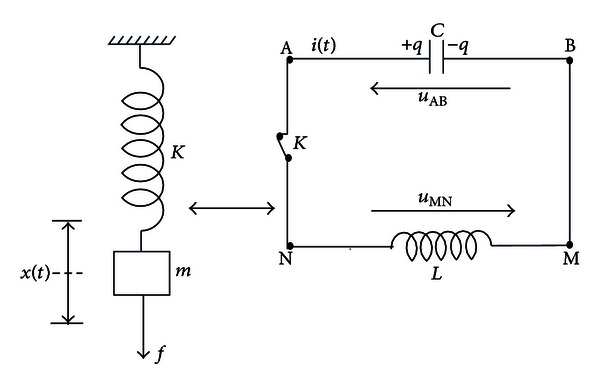
Electrical and mechanical resonant oscillators.

As shown by Figure 5, the mechanic oscillator with mass “*m*” and spring constant “*K*” is equivalent to electrical oscillator consists by a serial “*LC*” circuit.

 This oscillator (Figure 5) is generally used either for generating signals at a particular frequency *f*_0_ or picking out a signal at a particular frequency *f*_0_ from a more complex signal [21, 22]:

(3) $$\begin{array}{c} \omega =\sqrt{\frac{1}{LC}}\;=\frac{1}{\sqrt{LC}},\;\omega =2\pi f_{0}. \end{array}$$

The dynamics of this system are described as follows:

(4) $$\begin{array}{c} \frac{d^{2}q}{{dt}^{2}}+\frac{1}{LC}q=0. \end{array}$$

The solution of this equation consists of a signal *q*(*t*) defined by

(5) $$\begin{array}{c} q\left(t\right)=Q\cos\left(\omega t+\varphi \right)=\;Q\cos\left(\phi \left(t\right)\right). \end{array}$$

The system's total energy *E* is the sum of the electrical (capacitive) energy and magnetic (inductive) energy which is given by

(6) $$\begin{array}{c} E=\frac{1}{2}\frac{q^{2}}{C}+\frac{1}{2}\;L{\left(\frac{dq}{dt}\right)}^{2}\Rightarrow E=\frac{1}{2}L\omega^{2}Q^{2} \end{array}$$

such as *ω* = *dϕ*(*t*)/*dt*.

Based on this analysis, the Teager-Kaiser operator *ψ* is defined as follows:

(7) $$\begin{array}{c} \psi \left[q\left(t\right)\right]={\left(\frac{dq(t)}{dt}\right)}^{2}-\;q\left(t\right)\cdot \frac{d^{2}q\left(t\right)}{{dt}^{2}}. \end{array}$$

In the approximate discrete form of this operator we discredited the time *t* in *n* points and then

(8) $$\begin{array}{c} \psi_{d}\left[q\left(n\right)\right]=q^{2}\left(n\right)-\;q\left(n+1\right)\cdot q\left(n-1\right). \end{array}$$

In some cases it is made known that the speech signal can be modeled as a linear combination of AM-FM signals. Then the speech signal can be expressed as follows:

(9) s(t)=a(t)cos(ω0t+φ)=  a(t)cos(ϕ(t))=a(t)cos[∫0tωi(τ)dτ+ϕ(0)],

where *a*(*t*) is the amplitude signal depending on a time and *ω*_*i*_(*t*) is the instantaneous frequency defined by *ω*_*i*_ = *dϕ*(*t*)/*dt*.

Once applying the TEO to the speech signal give up

(10) $$\begin{array}{c} \psi \left[s\left(t\right)\right]≃{\left(a\left(t\right)\cdot \frac{d\phi \left(t\right)}{dt}\right)}^{2}. \end{array}$$

Herein, it is shown that TEO can track the modulation energy and identify the instantaneous amplitude and frequency. Motivated by this fact and in order to compute the real signal energy, we will use the TEO model as an alternative of using the commonly used instantaneous energy that only takes into account the “*s* ^2^” of the signal's source. The idea of using TEO is motivated by advantage of the modulation energy tracking capability of this technique. Indeed, the Teager-Kaiser estimated energy incorporates both amplitude and frequency information. It is hoped that additional information of the estimated energy will lead to an improvement of the accuracy of the automatic identification of pathological voices [23].

#### 2.5.3. Auditory Filter Bank (Gammatone)

**Figure 6:**
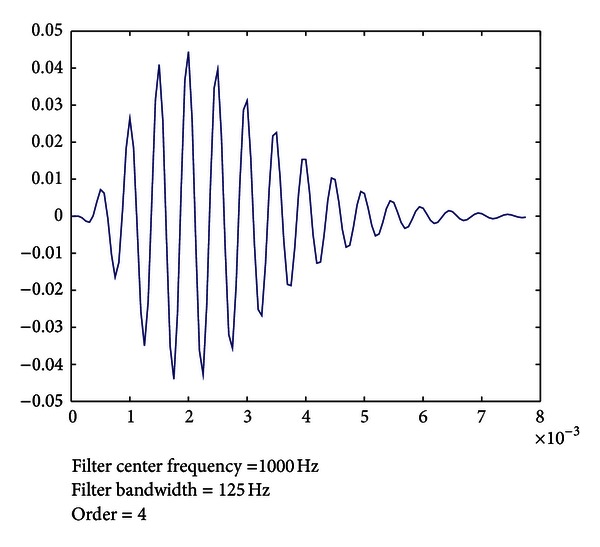
Gammatone function of the cochlear filter.

In auditory modelling, the digital filter bank is one of the most fundamental concepts that resemble the characteristics of the basilar membrane. In the inner ear's cochlea, each band-pass filter modeled response of part of the basilar membrane to some localized frequency information of the speech signals. Human auditory processing is based on a set of density frequency asymmetric filters used to estimate the activity of each frequency band. The bandwidth of asymmetrical filters is quantified using the notion of the Equivalent Rectangular Bandwidth (ERB). The Gammatone function that represents the impulse response of each filter has the following temporal form [9–11]:

(11) $$\begin{array}{c} g\left(t\right)=At^{n-1}\exp\left(-2\pi b\text{ERB}\left(f_{c}\right)t\right)\cos\left(2\pi f_{c}t\right), \end{array}$$

where *A*, *b*, *n* are the Gammatone filter design parameters and *f*_*c*_ is the center frequency of the filter.  Figure 6 shows the Gammatone function corresponding to a cochlea filter at order 4, centred at the frequency 1000 Hz and with bandwidth of 125 Hz.

In the Gammatone filter bank, the bandwidth of each filer is established according to the auditory critical band related to its centre frequency. Particularly, the filter's ERB is defined in Hz as in (12) and this is when we specified the magnitude of a filter's frequency response |*H*(*f*)| and the filter's maximum gain |*H*(*f*_max_)| at the frequency *f*_max_:

(12) $$\begin{array}{c} \text{ERB}=\frac{\int {\left|H(f)\right|}^{2}}{{\left|H(f_{\max})\right|}^{2}}. \end{array}$$

The ERB is the equivalent bandwidth of an orthogonal filter with constant gain |*H*(*f*_max_)| and energy equal to the original filter's energy. Such as the filter's energy is defined as the integral of the filter's frequency response squared. Based on the human physiology states, it is revealed in the current research [23] that the auditory filter bandwidths are given by the following ERB(*f*) function:

(13) $$\begin{array}{c} \text{ERB}\left(f\right)=6,23{\left(\frac{f}{1000}\right)}^{2}+93,39\left(\frac{f}{1000}\right)+28,52, \end{array}$$

where *f* is the filter center frequency expressed in Hz.

Using the critical Bark frequency scale, the filter insertion is equidistant as follows:

(14) $$\begin{array}{c} \text{Bark}\left(f\right)=\frac{26,81f\;}{f+3920}-\;0,53. \end{array}$$

Being given the sampling frequency of the signal, the frequency *f* must verify the condition 0 ≤ *f* ≤ *F*_*s*_/2.

Regarding (11) and taking the values *b* = 1,019 and *n* = 4 of auditory filters [24], as a result, the filter frequency response *G*(*ω*) is specified by

(15) G(ω)=A2  6  (2πb ERB(fc)+j(ω−ωc))4+A2  6  (2πb ERB(fc)+j(ω+ωc))4.

Taking into consideration that |*H*(*ω*_*c*_)| = 1, the filter gain *A* is situate and is equal to

(16) $$\begin{array}{c} A=\frac{1}{\sum_{k=1}^{N}t^{n-1}\exp\left(-2\pi b\;\text{ERB}\left(f_{c}\right)t\right)}, \end{array}$$

where *N* is the sample number of the impulse response. 

In anther study [25], the authors discussed two parameters to create a family of Gammatone filter banks. These parameters are the filter bank density (number of filters) and the filters bandwidth parameter denoted *F* which is a multiplier parameter (*F*∗ERB(*f*)). The results provided show that both parameters are important for robust speech recognition. Best results are obtained for *F* around 1.5 and for 30 filters.

**Figure 7:**
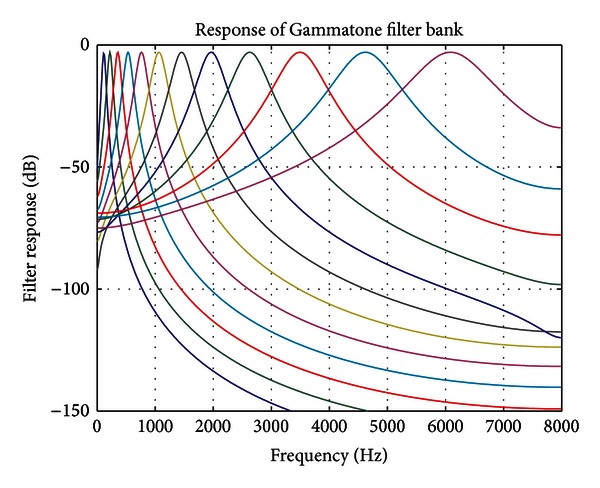
Gammatone filter bank with 25 filters.

Figure 7 shows an example of the Gammatone filter bank with 25 filters and with 1.5∗ERB(*f*).

## 3. Experiments and Results

### 3.1. Experimental Selection

We investigate the robustness of auditory TECCs (GTECCs) in noise by artificial addition of various levels of white noise to the speech signal and computing the correct classification rate (CCR) for each of MFCCs, MTECCs, and GTECCs features. The results are obtained using the databases described previously and based on the general classification algorithm shown in Figure 1 and on the block diagram of feature extraction shown in Figure 3. Concerning the development of the multilayer perceptron (MLP) and accordingly, the number of input layer nodes represents the number of voice quality features, while the single output layer nodes represent the two different class categories (pathological or normal). Many experimental investigations are conducted. The selected number of voice features is 13 MFCCs or TECCs. For the extraction of Gammatone Teager Energy Cepstrum Coefficients (GTECCs) we truncate the cepstrum coefficients to keep the first 13 coefficients similarly to the “standard” MFCC front end. The respective number of hidden nodes that provided the optimal result is 10 hidden nodes. Therefore, the architecture of the network is 13-10-1. The target mean square error (MSE) is fixed to 0.0001 after 5000 iterations. We have created the “Noisy Database” by adding white noise to the speech databases, respectively, at SNR levels of 0 dB, 5 dB, 10 dB, and 15 dB. We performed the CCR for each feature which is the average of two separate values corresponding to the two speech databases experiments. The 75% of the speech database was used to the training process, while the 25% was used to the validation (test) process.

### 3.2. Results and Discussion

The performance of each voice feature is performed using the correct classification rate (CCR). The speech database is mixed of pathological and normal voices, so the CCR is defined as follows:

(17) $$\begin{array}{c} \text{CCR}=\frac{{\text{CCR}}_{\left(\text{Normal}\right)}+{\text{CCR}}_{\left(\text{Pathological}\right)}}{2}, \end{array}$$

where

(18) $$\begin{array}{c} {\text{CCR}}_{\left(\text{Normal}\right)}=\frac{\text{Number}\;\text{of}\;\text{correct}\;\text{classification}\;\text{normal}\;\text{voices}\;}{\text{Total}\;\text{number}\;\text{of}\;\text{normal}\;\text{voices}}*100,\\ {\text{CCR}}_{\left(\text{Path}\right)}=\frac{\text{Number}\;\text{of}\;\text{correct}\;\text{classification}\;\text{pathological}\;\text{voices}\;}{\text{Total}\;\text{number}\;\text{of}\;\text{normal}\;\text{voices}}*100. \end{array}$$

The signal-to-noise ratio (SNR) is defined as

(19) $$\begin{array}{c} \text{SNR}=10\log\frac{\sum_{n=0}^{M-1}s^{2}\left[n\right]}{\sum_{n=0}^{M-1}n^{2}\left[n\right]}=10\log\frac{\sigma_{s}^{2}}{\sigma_{n}^{2}}, \end{array}$$

where *s* ^2^[*n*] and *n* ^2^[*n*] are, respectively, the speech and noise samples of analysed signal segment. Furthermore, *σ*_*s*_ and *σ*_*n*_ are, respectively, the power in the signal or noise frame.

Table 1 recapitulates the experiment results. It gives the CCRs for each voice feature: MFCC, MTECC, and GTECC at clean or noisy condition. In the noisy environment, the speech signal is admixed with white noise for different SNR levels.

**Figure 8:**
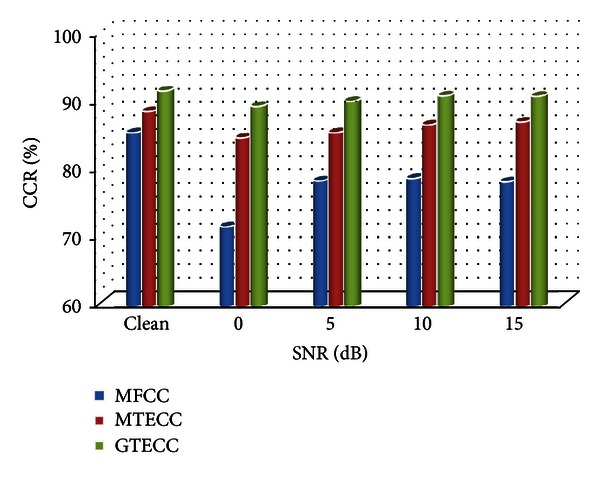
Feature performance in clean and noisy condition.

Table 1 presents the performance of three voice features in presence of various levels of additive noise. We note that the GTECC features that are extracted using the Gammatone filter bank exhibit the best CCR. Also, it is observable that the performance of the MFCC features decreases when the SNR decreases too, that is, when the speech signal becoming more noisy. Similarly, the performance of MTECC shows a decrease, but it is a relatively small decrease, whereas the GTECC features have the overall highest recognition rate throughout all SNR levels. These results assert well the major interest of the Teager energy operator and of the auditory filter bank analysis.  Figure 8 is a graphical representation of Table 1 results.

## 4. Conclusion

In this paper, we concentrated on the implementation of an automatic classification of pathological and normal voices system able to worke in noisy environments. This system uses Teager energy cepstral features extracted from an audio signal after analysis by Gammatone filter bank. The proposed features (GTECCs) have been shown to be more robust than MFCCs in white noise environments for low SNR values. For clean conditions and white noise, the MTECCs performed similarly to the GTECCs. In noisy environment, the MFCCs have the lowest classification accuracy but in clean condition there is no big difference with respect to TECC features. The increased robustness of GTECCs is due to both the auditory filter bank design and the Teager energy estimation. In fact, the Gammatone filter bank with filters placed according to the Bark scale and with bandwidths given by the ERB(*f*) is a good approximation of the human auditory system. Also, the TEO presents a demodulation-like operation and the envelope of the spectrum produces more robust features.

## Figures and Tables

**Table 1:** Feature performance in clean and noisy condition.

SNR (dB)	Clean	15	10	5	0
MFCC					
CCR_Norm_	86.76	80.52	80.22	78.59	72.06
CCR_Path_	84.13	77.32	77.36	78.29	71.43
CCR	85.45	78. 29	78.79	78.44	71.74

MTECC					
CCR_Norm_	88.24	86.76	87.50	86.76	85.29
CCR_Path_	88.89	87.30	85.71	84.13	84.13
CCR	88.56	87.03	86.61	85.45	84.71

GTECC					
CCR_Norm_	92.65	92.65	91.18	91.18	89.71
CCR_Path_	90.48	88.89	90.48	88.89	88.89
CCR	91.56	90.77	90.83	90.03	89.30

## References

[1] Yu P, Ouaknine M, Revis J, Giovanni A (2001). Objective voice analysis for dysphonic patients: a multiparametric protocol including acoustic and aerodynamic measurements. *Journal of Voice*.

[2] Boyanov B, Hadjitodorov S (1997). Acoustic analysis of pathological voices: a voice analysis system for the screening and laryngeal diseases. *IEEE Engineering in Medicine and Biology Magazine*.

[3] Parsa V, Jamieson DG (2000). Identification of pathological voices using glottal noise measures. *Journal of Speech, Language, and Hearing Research*.

[4] Dubuisson T, Dutoit T, Gosselin B, Remacle M (2009). On the use of the correlation between acoustic descriptors for the normal/Pathological voices discrimination. *EURASIP Journal on Advances in Signal Processing*.

[5] d’Alessandro C, Bozkurt B, Doval B (2007). Phase-based methods for voice source analysis. *Advances in Nonlinear Speech Processing*.

[6] Boashash B (1992). Estimating and interpreting the instantaneous frequency of a signal. I. Fundamentals. *Proceedings of the IEEE*.

[7] Liu L, He J, Palm G (1997). Effects of phase on the perception of intervocalic stop consonants. *Speech Communication*.

[8] Hegde RM, Murthy HA, Gadde VRR (2007). Significance of the modified group delay feature in speech recognition. *IEEE Transactions on Audio, Speech and Language Processing*.

[9] Ghitza O (1994). Auditory models and human performance in tasks related to speech coding and speech recognition. *IEEE Transactions on Speech and Audio Processing*.

[10] Glasberg BR, Moore BCJ (1990). Derivation of auditory filter shapes from notched-noise data. *Hearing Research*.

[11] Irino T, Patterson RD (1997). A time-domain, level-dependent auditory filter: the gammachirp. *Journal of the Acoustical Society of America*.

[12] Kortelainen J, Noponen K (2005). *Neural Networks*.

[13] Bishop CM (1996). *Neural Networks for Pattern Recognition*.

[14] Cherif A (2001). Pitch detection and formant extraction of Arabic speech processing. *Journal of Applied Acoustics*.

[15] Salhi L, Talbi M, Abid S, Cherif A (2011). Performance of wavelet analysis and neural networks for pathological voices identification. *International Journal of Electronics*.

[16] Kacha A, Grenez F, Schoentgen J (2006). Multiband frame-based acoustic cues of vocal dysperiodicities in disordered connected speech. *Biomedical Signal Processing and Control*.

[17] Bettens F, Grenez F, Schoentgen J (2005). Estimation of vocal dysperiodicities in disordered connected speech by means of distant-sample bidirectional linear predictive analysis. *Journal of the Acoustical Society of America*.

[18] Davis SB, Mermelstein P (1980). Comparison of parametric representation for monosyllabic word recognition in continuously spoken sentences. *IEEE Transactions on Acoustics, Speech, and Signal Processing*.

[19] Potamianos A, Maragos P (2001). Time-frequency distributions for automatic speech recognition. *IEEE Transactions on Speech and Audio Processing*.

[20] Murty KSR, Yegnanarayana B (2006). Combining evidence from residual phase and MFCC features for speaker recognition. *IEEE Signal Processing Letters*.

[21] Jabloun F, Çetin AE, Erzin E (1999). Teager energy based feature parameters for speech recognition in car noise. *IEEE Signal Processing Letters*.

[22] Kaiser JF (1983). Some observations on vocal tract operation from a fluid flow point of view. *Vocal Fold Physiology: Biomechanics, Acoustics, and Phonatory Control*.

[23] Patil HA, Parhi KK Novel variable length teager energy based features for person recognition from their hum.

[24] Kaiser JF On a simple algorithm to calculate the ‘energy’ of a signal.

[25] Dimitriadis D, Maragos P, Potamianos A Auditory teager energy cepstrum coefficients for robust speech recognition.

